# Oxygen Absorption in Electrocatalyst Layers Detected by Scanning Electrochemical Microscopy

**DOI:** 10.1002/celc.202100702

**Published:** 2021-08-16

**Authors:** Mahdi Moghaddam, Pekka Peljo

**Affiliations:** ^1^ Research Group of Physical Electrochemistry and Electrochemical Physics Department of Chemistry and Materials Science Aalto University Kemistintie 1, PO BOX 16100 00076 Aalto Finland; ^2^ Research Group of Battery Materials and Technologies Department of Mechanical and Materials Engineering University of Turku Turku 20014 Turun Yliopisto Finland

**Keywords:** Nafion, oxygen confinement, redox chemistry, scanning electrochemical microscopy, supported catalysts

## Abstract

Scanning electrochemical microscopy (SECM) is able to track the local electrochemical activity of an electrolyte‐immersed substrate employing an ultra‐micro‐electrode (UME) in micrometer‐scale spatial resolution. In this study, SECM is employed to investigate the presence of oxygen in the electrocatalyst layers of polymer electrolyte membrane fuel cells and electrolyzers. Approach curves on electrocatalyst layers with the tip potential set for oxygen reduction reveal that a significant amount of oxygen is absorbed in the catalyst layer. We confirm that the coexistence of Nafion ionomer and carbon black leads to oxygen confinement. It is suggested that this oxygen is confined within the hydrophobic parts of the self‐assembled Nafion on the graphitic surfaces of the carbon black.

## Graphical abstract

**Absorption of oxygen into the electrocatalyst layer** only in the presence of Nafion (Nafion+Catalyst) is confirmed using scanning electrochemical microscopy. This is justified from a positive feedback when the tip approaches the layer containing both catalyst and Nafion. In contrast, a negative feedback is observed for both the catalyst and Nafion, when tested separately, indicating no significant amount of oxygen is absorbed into either of them.

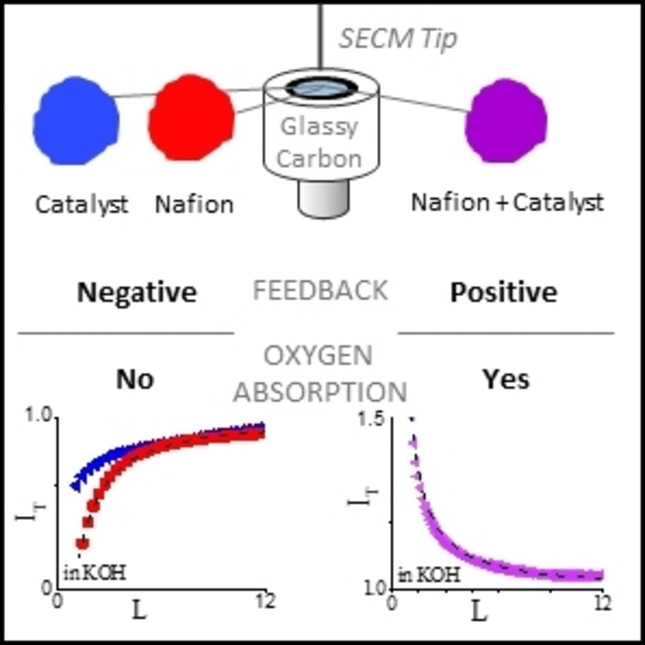

## 1 Introduction

Scanning electrochemical microscopy (SECM), as a powerful scanning probe technique, is able to track the local electrochemical activity of an electrolyte‐immersed substrate, as well as liquid/liquid and liquid/gas interfaces, employing an ultra‐micro‐electrode (UME) in the immediate vicinity of an interface. Micrometer scale spatial and millisecond scale temporal resolution is obtained with typical microelectrodes. SECM has been utilized in various applications from biology[^1^, ^2^] to energy devices.^[3]^

Thus far, SECM has been widely used to investigate the electrocatalytic activities of various catalysts for both hydrogen oxidation (HOR) and oxygen reduction (ORR) reactions in different modes, including “Tip Generation Substrate Collection“ (TG‐SC), “Substrate Generation Tip Collection“ (SG‐TC), and Feedback modes.[^4^, ^5^, ^6^, ^7^, ^8^, ^9^, ^10^, ^11^, ^12^, ^13^] In this work, we employ SECM in the feedback mode to investigate the presence of oxygen in the electroactive catalyst layers of polymer electrolyte membrane fuel cells (PEMFCs).

Incorporation of Nafion ionomer in PEMFCs electrodes was firstly reported by Raistrick more than 30 years ago. Their cell with Nafion impregnated electrodes was as efficient as a cell containing a 10 times higher loading of Pt in the catalyst with no Nafion on the electrodes.^[14]^ Later, Srinivasan *et. al* attributed this performance enhancement to the extension of three dimensional reaction zone, which is the contact of catalyst, reactants and the electrolyte, by Nafion.^[15]^ Over the last 30 years, numerous research contributions have been made regarding the effect of Nafion incorporation into the electrodes of energy devices, mostly PEMFCs, on the enhancement of ORR and/or HOR, and the overall cell performance. Today the utilization of Nafion is widespread in efficient electrodes along with its prominent application as the electrolyte.

Nafion ionomer exhibits a self‐assembly behavior in aqueous solutions.[^16^, ^17^, ^18^] This self‐assembly, on the catalyst layers of PEMFC electrodes, ensures sufficient ionic conductivity when the optimum amount of ionomer is present.[^19^, ^20^, ^21^] The properties, structure, and behavior of a self‐assembled thin layer of Nafion on various substrates has also been investigated in detail.[^19^, ^22^, ^23^, ^24^, ^25^] Chlistunoff and Sansiñena[^22^, ^26^, ^27^] published a series of contributions on ORR taking place on carbon‐supported catalysts in which they placed great emphasis on the role of Nafion self‐assembly by means of cyclic voltammetry and rotating ring disc electrode (RRDE) methods in an aqueous media. They demonstrated that Nafion self‐assembles through its hydrophobic component on the graphitic surfaces of the catalyst's carbon support. This could lead to a spillover of self‐assembled ionomers onto the active sites of the catalysts, namely Pt or macrocyclic compounds, and build obstacles to the ORR by blocking the pathways.^[26]^ Furthermore, they discussed the novel concept of oxygen confinement within the self‐assembled Nafion.[^22^, ^27^] They reported that oxygen could be confined within the interface of the hydrophobic part of the self‐assembled ionomer and the graphitic surfaces of the catalyst's carbon support in aqueous media. In the present work, the confinement of oxygen within the catalyst layers is investigated using the SECM technique in feedback mode.

In the SECM feedback mode, the biased UME tip vertically approaches the substrate and the redox mediator of interest present in the solution (oxygen in this work) is reduced at the tip. When the tip travels within the “bulk solution“ (∼d>10a, where d is the tip‐to‐substrate distance and a is the tip electroactive radius^[28]^ ), there is a steady‐state reduction current at the tip due to the hemispherical diffusion profile of microelectrodes. When the tip is in the vicinity of the inactive substrate, the closer the tip is to the substrate, the less diffusion volume is available for the redox mediator in the solution. Due to this substrate blockage, the reduction current at the tip decreases relative to the steady‐state current, as the tip becomes closer to the substrate. This current response is called “negative feedback“ and customarily is displayed in a distance‐current curve known as the SECM “approach curve“. When the tip approaches an active substrate, perturbation of the redox equilibrium by the tip leads to a local decrease in the Nernst potential of the solution.^[29]^ However, the redox potential of the sufficiently conductive and electroactive substrate remains equilibrated with the electrolyte potential determined by the Nernst potential of the bulk solution. This leads to a situation where the substrate feels a “mixed potential”,^[30]^ where the current flows through the substrate to equilibrate the Nernst potential of the electrolyte over the whole substrate surface. Now, all the reduced species diffusing from the tip are oxidized on the substrate, leading to a “positive feedback“ response through which the reduction current at the tip increases relative to the current in the bulk solution. Slow kinetics or poor conductivity of the substrate can lead to a mixed response.

When approaching an interface, the tip that is biased for oxygen reduction reaction perturbs the oxygen partition equilibrium across the interface by consuming (reducing) the oxygen. To equilibrate the chemical potentials of oxygen in the solution and across the interface, oxygen partitioning takes place and additional oxygen is supplied from across the interface, or from within the porous interface. For example, an oxygen partition induced positive feedback is observed when approaching a liquid‐air interface,^[31]^ a water‐air interface,[^32^, ^33^] or an interface with a porous hydrophobic film.^[1]^ In these cases, the positive feedback is not due to the electrochemical reaction on an electrochemically active substrate, but positive feedback due to the availability and partitioning of the additional oxygen for equilibrating the chemical potentials of the oxygen in the solution and across the interface. In this work, as a similar approach, SECM is used to perturb the oxygen partition equilibrium over the porous electrocatalyst layers containing Nafion to study the absorbed oxygen within the electrocatalyst layers.

**Figure 1:**
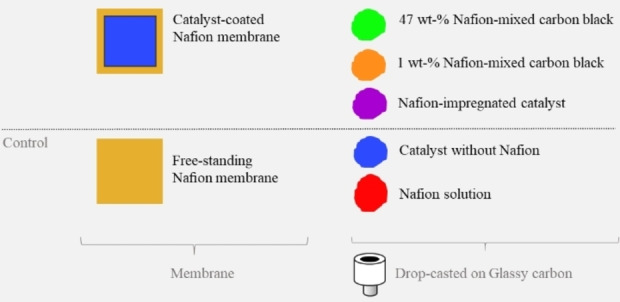
Seven various substrates used for the SECM experiment (colours are in harmony with Figures 3 and 4).

To investigate the effect of Nafion, different approaches were taken. In two substrates, the catalyst (20 wt‐% Pt on carbon black) was in contact with Nafion. Firstly, the catalyst ink was spray‐coated on the Nafion 115 membrane, as described by Sorsa *et al*.^[34]^ Secondly, the Nafion ionomer solution was drop‐casted on a dried porous catalyst layer, which had already been drop‐casted on a glassy carbon. Two other SECM substrates were also used, C65 carbon black ink without any catalyst particles was mixed with Nafion in two different ratios of 1 wt‐% and 47 wt‐% and then drop‐casted on the glassy carbon. In addition, control experiments were performed with a free‐standing Nafion membrane, a drop‐casted Nafion ionomer and a drop‐casted catalyst ink (without Nafion) on clean glassy carbon electrodes (refer to Experimental Section for detailed procedures). Therefore, in total, seven various substrates were selected for the SECM experiment (Figure 1).

**Figure 2:**
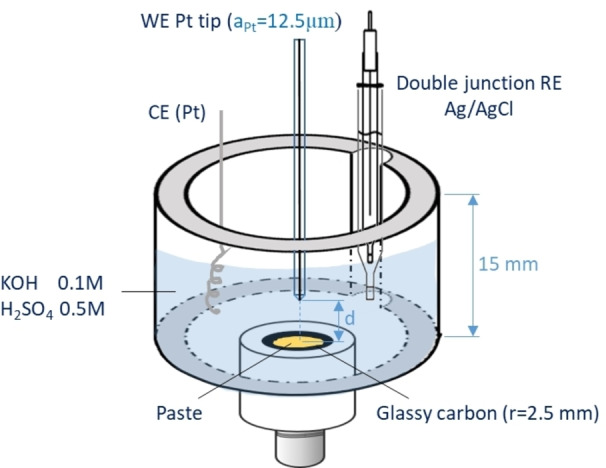
SECM setup.

The substrates were experimented on using the SECM setup shown in Figure 2 (refer to Experimental Section for details).

## 2 Results and Discussion

**Figure 3:**
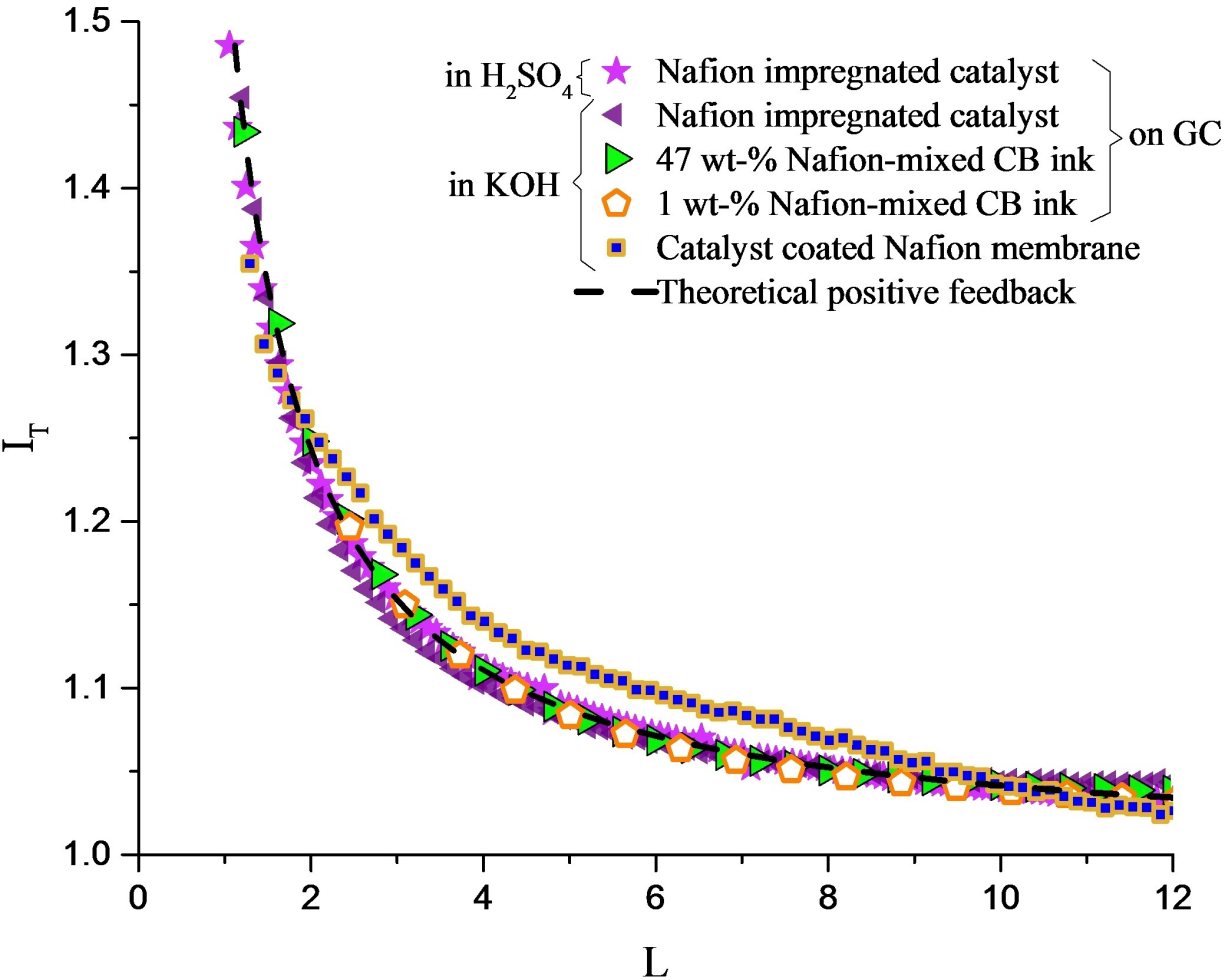
Current‐distance response of the Pt tip approaching Nafion impregnated catalyst in H_2_SO_4_ ( 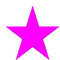) and in KOH ( 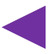), and 47 wt‐% Nafion‐mixed ( 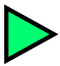) and 1 wt‐% Nafion‐mixed ( 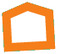) carbon black inks in KOH on glassy carbon, and catalyst coated Nafion membrane ( 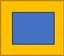) in KOH. The dashed line represents the theoretical positive feedback.

**Figure 4:**
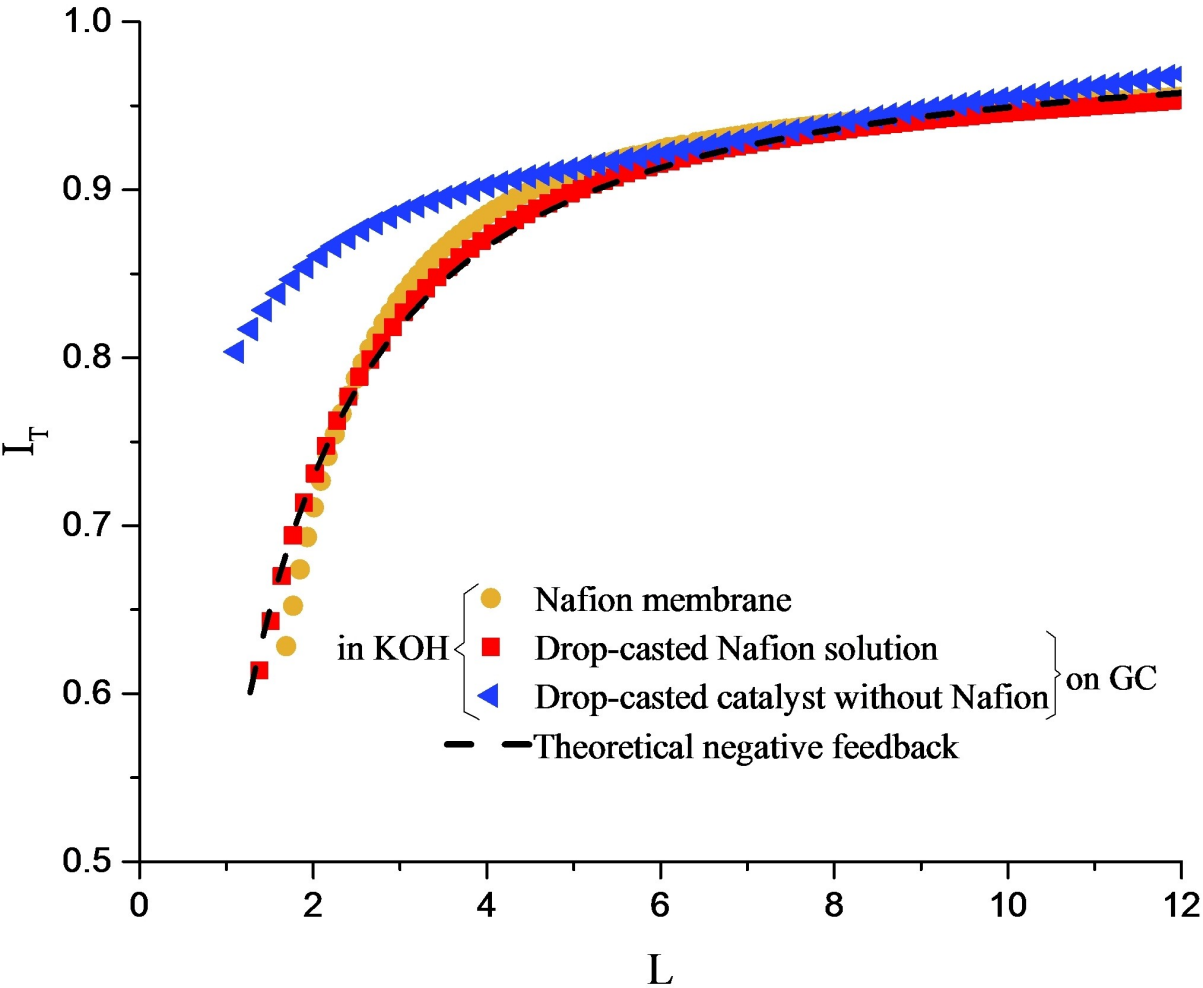
Current‐distance response of the Pt tip in KOH electrolyte approaching a free‐standing Nafion membrane ( 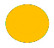), a drop‐casted Nafion solution on glassy carbon ( 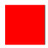), and a drop‐casted catalyst without Nafion on glassy carbon ( 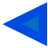). The dashed line represents the theoretical negative feedback.

Approach curves in Figures 3 and 4 show the normalized UME tip current, *I*_T_, vs. the normalized tip distance from the substrate, *L. I*_T_ is defined as *i*_T_/*i*_T∞_ where *i*_T_ is the measured reduction current at the tip and *i*_T∞_ is the steady‐state current measured in the bulk solution. *L* is defined as “*d*/*a*“, where d is the tip‐to‐substrate distance and a is the tip electroactive radius, defined in Figure 2. The dashed lines in Figure 3 and Figure 4 represent the theoretical positive and negative feedbacks, respectively.^[35]^ The empirical equations for these curves are shown in the SI.

As shown in Figure 3, the Nafion impregnated catalyst and Nafion‐mixed carbon black inks drop‐casted on glassy carbon, exhibit positive feedback response, and the same result is obtained with the catalyst coated Nafion membrane. The curves display a general agreement with the curve for the theoretical positive feedback, although deviations in medium *L* values are observed in catalyst coated Nafion membrane. Two factors might give rise to this deviation. Firstly, the membrane is not fixed as in the other experiments with glassy carbon as the substrate; therefore, it may not be completely horizontal. Secondly, on a microscopic scale the membrane might include some waves in the surface that affect the solution interaction volume while the tip is a few microns away from the membrane.

Approach curves for the Nafion ionomer and Nafion‐free catalyst ink drop‐casted on a glassy carbon electrode, and for the Nafion membrane are shown in Figure 4, displaying a negative feedback. Approach curves for pure Nafion (solution and membrane) show excellent agreement with the theoretical curve for negative feedback, and the same behavior is observed for clean glassy carbon electrode (results not shown). The drop casted catalyst solution without Nafion shows an intermediate behavior between the positive and negative feedbacks. Nevertheless, while approaching the substrate, the tip current decreases to lower values than the limiting current. To prevent damage to the tip, the approach was interrupted before the tip crashed into the surface. Therefore, the tip distance from the substrate at the end of the approach curve is not known precisely and the normalized distance (*L*) in figures 3 and 4 is an estimation.

As the tip is biased for the oxygen reduction potential, any current increase while the tip is approaching the substrate is attributed to the oxygen in excess of the solvated oxygen in the electrolyte. In this way, positive feedback indicates that there is an excess of oxygen absorbed in the catalyst layers. In contrast, when the tip current decreases as the tip approaches the substrate, the solvated oxygen would be the only source for the tip current, and the substrate would act as a block for oxygen diffusion to the tip. Therefore, negative feedback indicates that no significant amount of oxygen is present in the substrate.

Based on the observed negative feedback response, it is therefore clear that pure Nafion does not significantly absorb oxygen. Moreover, the drop‐casted catalyst on glassy carbon without the presence of Nafion appears to be unable to confine large amounts of oxygen within its structure as in its mixed response the tip current still decreases to lower values than the limiting current while approaching the substrate (Figure 4).

On the other hand, the Nafion impregnated catalyst shows a positive feedback response in both acidic and basic environments. The catalyst layer coated Nafion membrane exhibits a positive feedback response as well. In other words, it appears that both Nafion and high‐surface area electrode are required for the presence of excess of oxygen.

An elementary assumption is that the oxygen trapped within the substrate during the coating, or the drop‐casting processes would be the source of excess of oxygen and a consequently positive feedback response. The trapped oxygen is a temporary (thermodynamically not stable) source for oxygen in the substrate and after it is depleted from the substrate, the positive feedback should not be observed anymore. To investigate this, the glassy carbon substrate was biased for the ORR potential (−0.6 V in KOH 0.1 M – refer to SI) so that any present oxygen within the substrate is reduced and the substrate is depleted from oxygen. During the ORR on the substrate the tip was located a few microns away from the substrates being biased for the same potential as the substrate (−0.6 V). The tip current for oxygen reduction reached zero, confirming that all the oxygen within the substrate along with the solvated oxygen in the solution in the vicinity of the substrate (the zone where the tip was located) was consumed during the ORR on the substrate. Next, the substrate was unbiased again and the current flow at the tip (biased for −0.6 V) was measured. The tip current increased gradually and after 30 minutes reached higher values than the limiting current (bulk current), similar to the observed increased currents in the approach curves. As the current increased to higher values than the bulk current (current due to the reduction of solvated oxygen far away from the substrate), some oxygen is again supplied from the substrate while the oxygen had been depleted previously from the substrate. It shows that the amount of oxygen in the substrate was recovered by diffusion and partitioning from the bulk electrolyte into the substrate in an equilibrium. Therefore, the oxygen entrapment assumption is not valid and instead oxygen is thermodynamically stable within the substrate.

As discussed earlier, it is suggested that oxygen can be confined (absorbed) in the hydrophobic part of the self‐assembled Nafion ionomer on the graphitic surfaces of the catalyst's support (carbon black).[^22^, ^27^] This could be the reason for the presence of excess of oxygen in the substrate and consequently the observed positive feedback response.

To investigate whether or not platinum particles are also required for oxygen confinement, carbon black without any catalyst particles was mixed with the Nafion solution in two different ratios and utilized as SECM substrate. As shown in Figure 3, Nafion‐mixed carbon black inks represent the positive feedback response. This indicates that the presence or absence of platinum particles in the substrate does not interfere with oxygen confinement. As a control experiment, Nafion solution was drop casted on a clean glassy carbon. The drop casted Nafion on glassy carbon showed the negative feedback response (Figure 4), indicating that no significant amount of oxygen is confined on the glassy surface.

### 2.1 Finite Element Simulations

To better understand the observed behavior, finite element simulations of the system were performed, as described in the SI. The excess oxygen in the electrode was described as an extra phase, with a partitioning coefficient of oxygen governing the equilibrium between the two phases. The other important parameters are the transfer rates of oxygen between these phases, as well as the oxygen diffusivity in this phase. This phase can be thought either as a gas phase, or as another phase of oxygen absorbed on the carbon surfaces. The simulations show that a positive feedback response is achieved with a sufficiently high partition coefficient (*ca*. 100 times more oxygen in the extra phase) and transfer rate (>100 s^−1^ from extra phase to electrolyte) when the diffusion coefficient of the oxygen in the water is used for oxygen in the extra phase. If the oxygen diffusion in the air is considered instead, a positive feedback response is also obtained with low partition coefficients (down to 1) if the transfer rate is very high (>1000 s^−1^).

We can now compare the approach curves obtained for the catalyst ink without Nafion, showing a mixed response, and 1 wt‐% Nafion containing carbon black ink showing positive feedback. A similar response is obtained in simulations if the partition coefficient and transfer rates are decreased, indicating that both the amount of excess oxygen and interfacial area available for the transfer between extra phase and electrolyte decrease in the absence of Nafion.

### 2.2 Oxygen Absorption in Electrocatalyst Layers: Future Studies

Electrocatalyst layers containing carbon and Nafion are widespread in electrochemical devices, from experimental methodologies to testing the catalysts with rotating disk electrode (RDE),^[36]^ and from lab‐scale cells[^37^, ^38^] to commercial fuel cells and electrolyzers. In electrocatalyst testing, for example under ORR conditions in the RDE, catalyst layer will continuously absorb oxygen from the solution. Therefore, the effective initial oxygen concentration within the catalyst layer would be much higher than the oxygen concentration expected for oxygen‐saturated solution. This may result in higher currents at low overpotential, especially if the potential is swept from high potentials to low potentials. At limiting currents where oxygen concentration in the catalyst layer reaches zero, there will be no effect due to absorbed oxygen. Therefore, RDE measurements showing hysteresis for different sweep directions indicate oxygen absorption in the catalyst layer. For fuel cells and electrolyzers, there is almost no aqueous phase, as water filled Nafion membrane functions as the electrolyte. In this study we have shown that excess oxygen is present also in the catalyst layer of a Nafion membrane immersed in an electrolyte solution, but it is not clear if the excess oxygen will be present if equilibrated with a gas phase. We believe that the answer is yes. When the catalyst layer become moist due to the generated H_2_O, there would definitely be excess oxygen present in the layer. If a fuel cell is fed with air, it becomes possible to reach a mass transfer limitation for oxygen. In such a case all the absorbed oxygen would also be reduced to water. For gas evolution reactions, oxygen would partition into the catalyst layer and reduce the oversaturation in the liquid phase, slowing down the bubble formation. Simulations taking these effects into account should be performed to determine, what would be the practical effect of oxygen absorption in the catalyst layer. Experimental comparison of different catalyst supports such as functionalized carbon, nanotubes, graphene, non‐carbon supports including TiO_2_, SiC, and other oxides^[39]^ could also be beneficial for evaluating if oxygen absorption in the catalyst layer plays a significant role on the fuel cell or electrolyzer performance.

Another interesting question is that if the absorption effect is only limited to the oxygen. We believe that other gasses such as hydrogen will show the same behavior, but this should be tested experimentally. In addition, how does the hydrophobicity of the catalyst layer contribute to the absorbed oxygen? And does this change under operation? We have shown that SECM is a good tool to measure these effects, but better quantification of the experimental results is required. This requires better knowledge of the structure of the electrocatalyst layer, including porosity. Additionally, operando spectroscopy measurements of the liquid and gas phase fractions would be beneficial, as at the moment these parameters remain open questions.

## 3 Conclusions

The SECM feedback mode is used to evaluate the Nafion induced oxygen confinement or absorption in electrocatalyst layers. The positive feedback response of the SECM test indicates that large amounts of excess oxygen is present in the substrate, while the negative feedback indicates the absence of additional oxygen. Due to the negative feedback response, Nafion in either form, the drop‐casted solution on glassy carbon, or the membrane, cannot confine the oxygen in its structure. Moreover, the drop‐casted catalyst on glassy carbon without the presence of Nafion is unable to confine significant amounts of oxygen. In contrast, the Nafion impregnated catalyst and the catalyst‐coated Nafion membrane exhibit a positive feedback response. Therefore, the oxygen is only present with the coexistence of the Nafion and the catalyst. It is suggested that the oxygen is confined within the hydrophobic part of the self‐assembled ionomer on graphitic surfaces of the catalyst's support, maintaining an equilibrium with the solvated oxygen in the electrolyte. The carbon black with two different ratios of mixed Nafion solution also presented a positive feedback response indicating that the presence or absence of Pt particles in the substrate does not interfere with Nafion self‐assembly and the consequent oxygen confinement.

## Experimental Section

### Chemicals

The catalyst, nominally 20 wt‐% Pt on carbon black was purchased from Alfa Aesar. The utilized carbon black was Timcal Super C65. Nafion 115 Dupont membrane was purchased from Ion Power and the 5 wt‐% Nafion solution was purchased from Sigma Aldrich. The Ultrapure Milli‐Q water (Millipore) and the 2‐propanol as solvent were used for ink preparation.

### Ink Preparation and Drop‐casting

The catalyst ink was prepared by adding 5 mg of 20 wt‐% Pt on carbon black to 1.3 ml of solvent containing 80 vol‐% water and 20 vol‐% 2‐propanol. The dispersion was stirred for 30 minutes followed by 15 minutes of sonication and another 30 minutes stirring to homogenize the ink. For drop‐casting, 5 μl of the ink was pipetted onto the glassy carbon (0.1 mg/cm^2^) and left to dry in the ambient atmosphere. A further 5 μl of the ink was dropped on top of the dried layer to reach a final concentration of 0.2 mg/cm^2^.

The carbon black ink was prepared by adding 4.4 mg of carbon black to 260 μl of solvent containing 20 vol‐% water and 80 vol‐% 2‐propanol. Next, the ink was mixed with Nafion in two different ratios of 1 wt‐% and 47 wt‐%. A similar method to that of drop‐casting the catalyst on glassy carbon was followed for Nafion‐mixed carbon black inks and the final concentration reached 0.86 mg/cm^2^ on glassy carbon.

### SECM Setup and Experiment

The SECM setup, shown in Figure 2, is composed of a PTFE cylindrical chamber with diameter and height of 15 mm, with a removable glassy carbon electrode with a radius of 2.5 mm (0.1 in) at the bottom. For the experiments with Nafion membranes, the membranes were placed in the same chamber so that the corners were in contact with the interior wall (the glassy carbon at the bottom was only used for sealing the chamber). Homemade silver/silver chloride double junction electrode and Pt wire were used as reference and counter electrodes, respectively. Potassium hydroxide 0.1 M or sulfuric acid 0.5 M were utilized as electrolytes. A homemade UME electrode, with a platinum radius of a=12.5 μm and glass sheath radius of 100 μm at the tip, was inserted into the electrolyte by means of a positioning system (CHI900 SECM, USA) as the working electrode. The cleanliness and the absence of scratches on the UME tip surface was assured by polishing with 0.05 μm alumina disk and optical microscopy, before and after each SECM experiment. The tip bias potential was selected prior to each approach curve measurement based on the cyclic voltammetry in the bulk solution so that diffusion limited oxygen reduction took place (refer to SI).

## Conflict of interest

The authors declare no conflict of interest.

## Supporting information

As a service to our authors and readers, this journal provides supporting information supplied by the authors. Such materials are peer reviewed and may be re‐organized for online delivery, but are not copy‐edited or typeset. Technical support issues arising from supporting information (other than missing files) should be addressed to the authors.

Supporting Information
