# Supplementary material for: Oxygen Absorption in Electrocatalyst Layers Detected by Scanning Electrochemical Microscopy
Source: ChemElectroChem. 2021 Aug 16;8(15):2950–5. doi: 10.1002/celc.202100702 (PMC8457140; doi:10.1002/celc.202100702)
Supplement: Supplementary file 1 — Supporting Information [file CELC-8-2950-s001.pdf]

ChemElectroChem

Supporting Information

Oxygen Absorption in Electrocatalyst Layers Detected by Scanning Electrochemical Microscopy

Mahdi Moghaddam and Pekka Peljo*

Contents

Finite element simulations of Scanning Electrochemical Microscopy 1

Absorbed oxygen diffusion as in the liquid phase 3

Absorbed oxygen diffusion as in the gas phase 7

Empirical equations for the theoretical positive and negative feedback response 11

Theoretical negative feedback (NI_T) is obtained with the empirical equation S7: 11

Cyclic voltammetry 11

Supplementary references 12

Finite element simulations of Scanning Electrochemical Microscopy

SECM simulations were performed as described in ref.[1] but in more detail to evaluate the effect of different parameters on the SECM response. Numerical finite element simulations of the system were performed with COMSOL Multiphysic software, v. 5.6. The catalyst layer was described as a porous electrode with a thickness of 20 µm. The different parameters used for the simulations are tabulated in Table S1.

Table S1. Parameters for the simulations

Parameter	Value	Notes
Tip radius	5 µm	Experimental
Radius of the insulating glass (RG)	8 × tip radius	Experimental
Diffusion coefficient of oxygen	1.93×10^{-5} cm^2/s (water) 0.176 cm^2/s (gas)	
Oxygen concentration in 0.1 M KOH	0.265 mM	Air-saturated solution
Porosity of the catalyst layer	varied from 0.1 to 0.9	Liquid volume fraction, assumption
Gas volume fraction	same as porosity or half the porosity	Assumption
Thickness of the catalyst layer	20 µm	Estimated from SECM measurements between catalyst and substrate covered parts

As before, the simulations were performed in 2-D axis symmetry with sufficiently large domain and mesh density to allow obtaining microelectrode limiting currents within 3 % of the theoretical value. "Transport of Diluted Species–physics" was utilized for the diffusion of all the species in aqueous phase and "Transport of Diluted Species in Porous Media–physics" was utilized for transfer of oxygen within the film. The porosity of the film was varied in the simulations from 0.2 to 0.8. Typically, catalyst inks drop-casted on glassy carbon show porosities up to 0.6[2], but obviously will vary depending the ink composition etc. The effect of diffusion coefficient of oxygen inside the catalyst layer was evaluated, as it could be considered that oxygen is trapped as gas, resulting in very high diffusivity. Therefore, simulations with oxygen diffusion coefficient in water and in gas phase were carried out. Two different approaches to porosity were considered: i) void fraction contains both the electrolyte and absorbed gas, or ii) void fraction is filled by equal amount of electrolyte and gas phase. The first approach is not realistic, as the gas and the electrolyte would share the same volume, but it can be considered as a first approximation. The second approach is more realistic, but the ratio of the electrolyte and gas phases is not known. Figure S1 shows the situation assuming porosity in the catalyst layer is 0.6 and half of the voids are filled with the electrolyte (blue) and other voids are filled with the gas phase (white). Moreover, the gas phase volume should change when oxygen is consumed. Therefore, more experimental data, for example with micro-x-ray tomography, would be required for accurate simulations. Nevertheless, comparison of the simulations and experiments will give insights into the real situation.

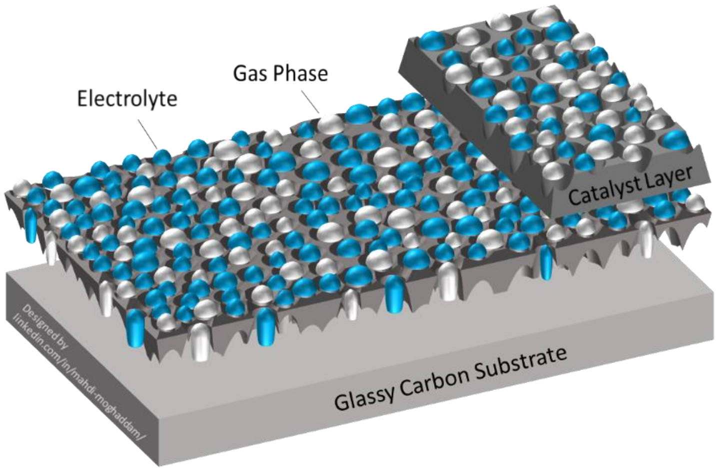

Figure S1. Drop casted catalyst layer on the glassy carbon. Porosity in the catalyst is 0.6, where half of the voids are filled with the electrolyte (blue), and other voids are filled with the gas phase (white).

The distance of the tip to the substrate was varied with a parametric sweep, and the tip current was evaluated in the steady-state. The equations have been described before in ref.[1]. Shortly, the general diffusion equation for a species *i* is:

$$\nabla \cdot \left(-D_i \nabla c_i\right) = R_i \tag{S1}$$

where *c* is concentration, *t* is time, *D* is the diffusion coefficient and *R* is the reaction term for the species *i*. For steady-state simulations, the time derivate is zero. The species in the aqueous phase are O_2 and OH^-. This oxygen absorbed in the catalyst layer, $O_2(g)$, is considered to be in equilibrium with the dissolved oxygen in the liquid phase:

$$\mathrm{O_2(aq)} \underset{k_{-1}}{\overset{k_1}{\rightleftarrows}} \mathrm{O_2(g)} \tag{S2}$$

with the equilibrium constant

$$K = \frac{c_{\mathrm{O_2(g)}}}{c_{\mathrm{O_2(aq)}}} = \frac{k_1}{k_{-1}} \tag{S3}$$

where k_1 is the rate constant to transfer O_2 from the aqueous phase into the gas phase and k_{-1} is the rate constant for the inverse reaction. As, discussed in ref.[1], partition coefficients of a couple of hundred are reasonable. Within the electrode the reaction term in Eq. S1 for oxygen transfer from gas phase to aqueous phase is:

$$R_{\mathrm{O_2(aq)}} = -R_{\mathrm{O_2(g)}} = k_{-1}\left[\mathrm{O_2(g)}\right] - k_1\left[\mathrm{O_2(aq)}\right] \tag{S4}$$

As discussed in ref.[1], this is a rough approximation of the actual interfacial reactions. According to the estimations in ref.[1], high surface area material of 1000 m^2/g could results in in apparent transfer rate of 1×10^6 s^{-1}. Carbon black has a surface area of *ca*. 250 m^2/g, and not all of this area will be available for gas transfer, but apparent transfer rates of up to 1×10^4 s^{-1} utilized in these simulations are still reasonable.

Bruggeman model was used to take into account the transport in the porous media, so that

$$D_\varepsilon = \frac{\varepsilon}{\tau} D, \quad \tau = \varepsilon^{-1/2} \tag{S5}$$

where D_ε is the diffusion coefficient in the porous media, ε is the porosity (the volume fraction of the phase where the diffusion takes place), and τ is the tortuosity.

The following electrode reaction takes place at the Pt tip at the diffusion limited rate:

$$O_2(aq) + 2H_2O + 4e^- \longrightarrow 4OH^-(aq) \quad \text{(S6)}$$

i.e. the surface concentration of O_2(aq) at the tip is 0. The flux of OH^- at this surface is then calculated from eq. S6 and the flux of O_2(aq) at the tip. The electrode/catalyst layer boundary is insulating for all species, and concentration boundary conditions of the bulk concentration are enforced at the outer boundaries of the geometry.

Absorbed oxygen diffusion as in the liquid phase

Figures S2 and S3 show the simulated approach curves with different values of K and k_{-1} and for porosity. The curves for both positive and negative feedback calculated with the theoretical expressions of Lefrou and Cornut[3] (dotted lines), considering only a flat substrate are shown for comparison.

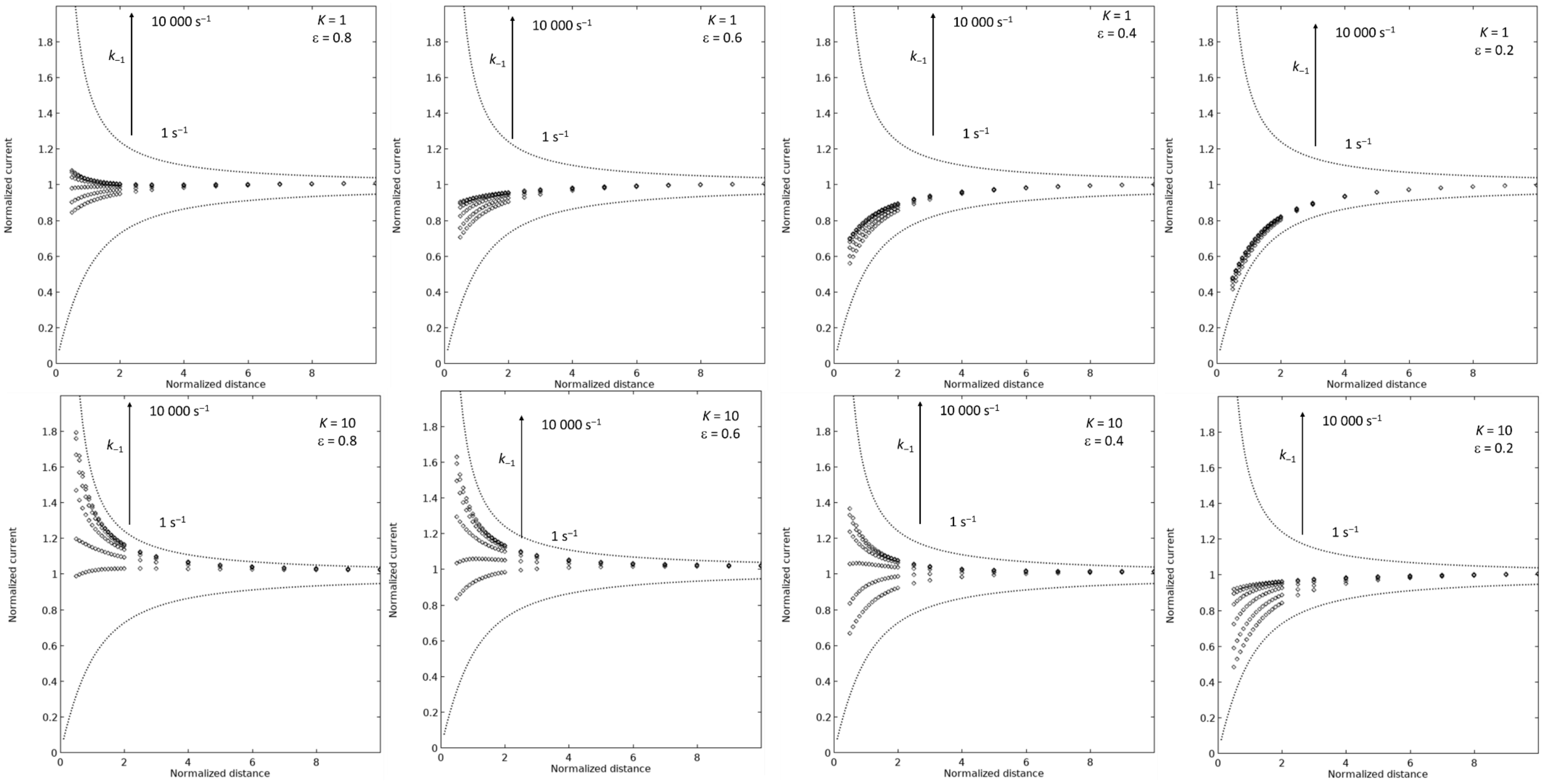

10 000 s^{-1}
k_{-1}
1 s^{-1}
K = 1
ε = 0.8
ε = 0.6
ε = 0.4
ε = 0.2
K = 10
Normalized current
Normalized distance

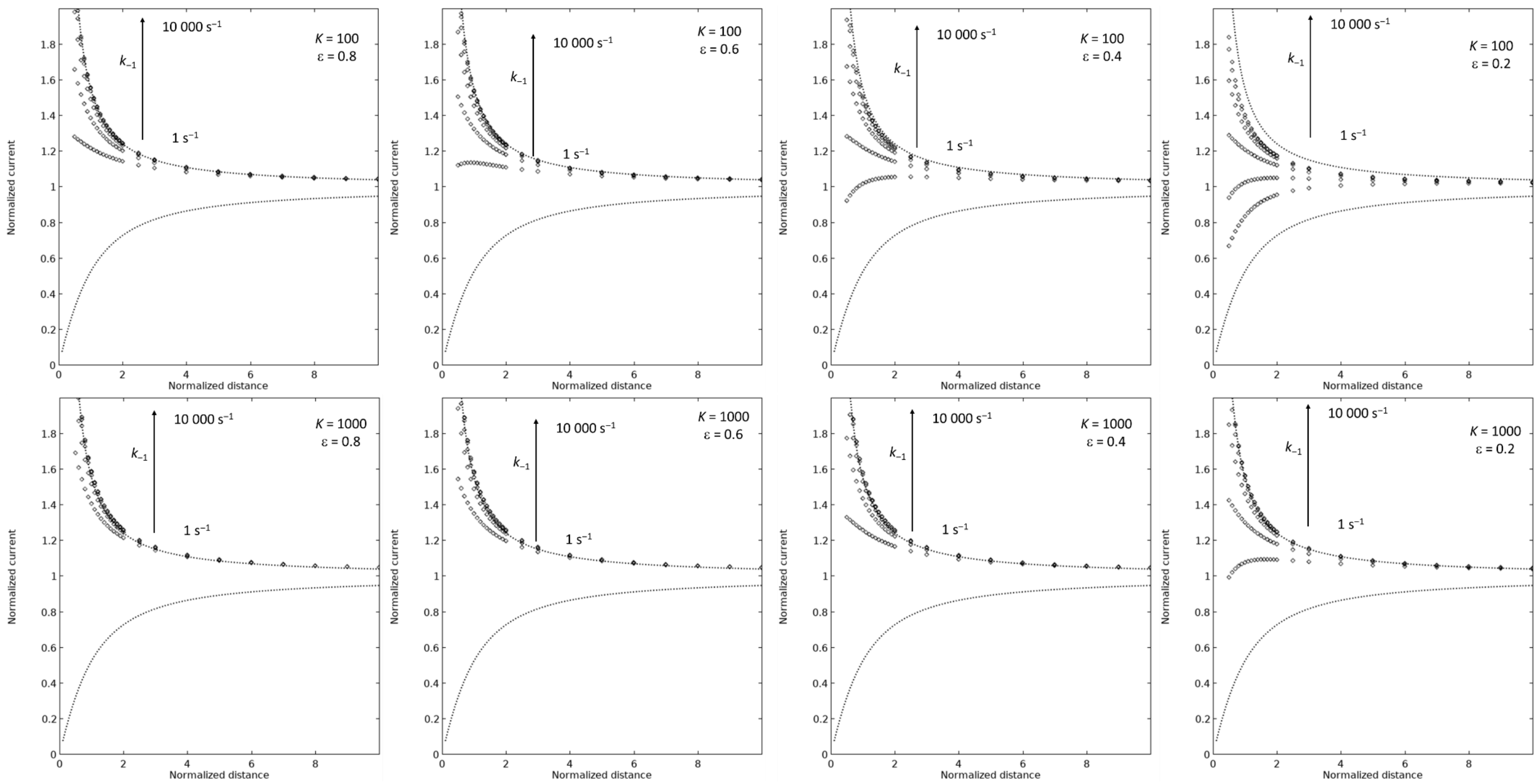

Figure S2. Simulated approach curves for electrode porosity *ε* from 0.2 to 0.8, partition coefficient *K* from 1 to 1000 and apparent transfer rate from gas phase to aqueous phase from 1 to 10 000 s^{-1} (steps of 1 order of magnitude). Diffusion coefficient of oxygen in the aqueous phase is utilized for excess oxygen absorbed in the catalyst layer. Absorbed oxygen and electrolyte share the same space.

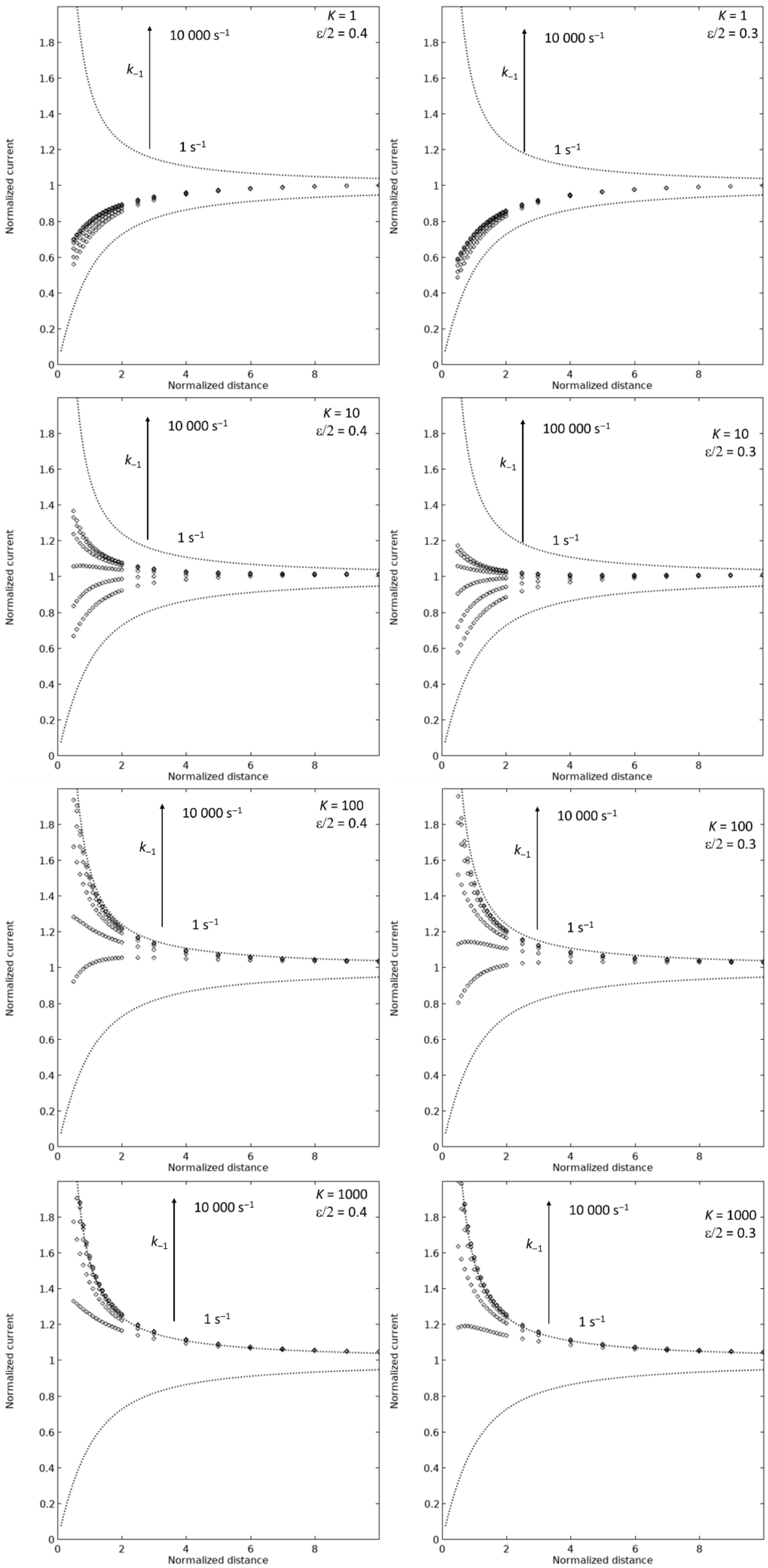

Figure S3. Simulated approach curves for electrode porosity *ε* from 0.2 to 0.8, partition coefficient *K* from 1 to 1000 and apparent transfer rate from gas phase to aqueous phase from 1 to 10 000 s^{-1} (steps of 1 order of magnitude). Diffusion coefficient of oxygen in the aqueous phase is utilized for excess oxygen absorbed in the catalyst layer. The volume fraction of the electrolyte phase is half the porosity of the electrode, and volume fraction of the gas phase is equal to the volume fraction of the electrolyte.

Absorbed oxygen diffusion as in the gas phase

Figures S4 and S5 show the simulated approach curves with different values of K and k_{-1} and for porosity. The curves for both positive and negative feedback calculated with the theoretical expressions of Lefrou and Cornut[3] (dotted lines), considering only a flat substrate are shown for comparison.

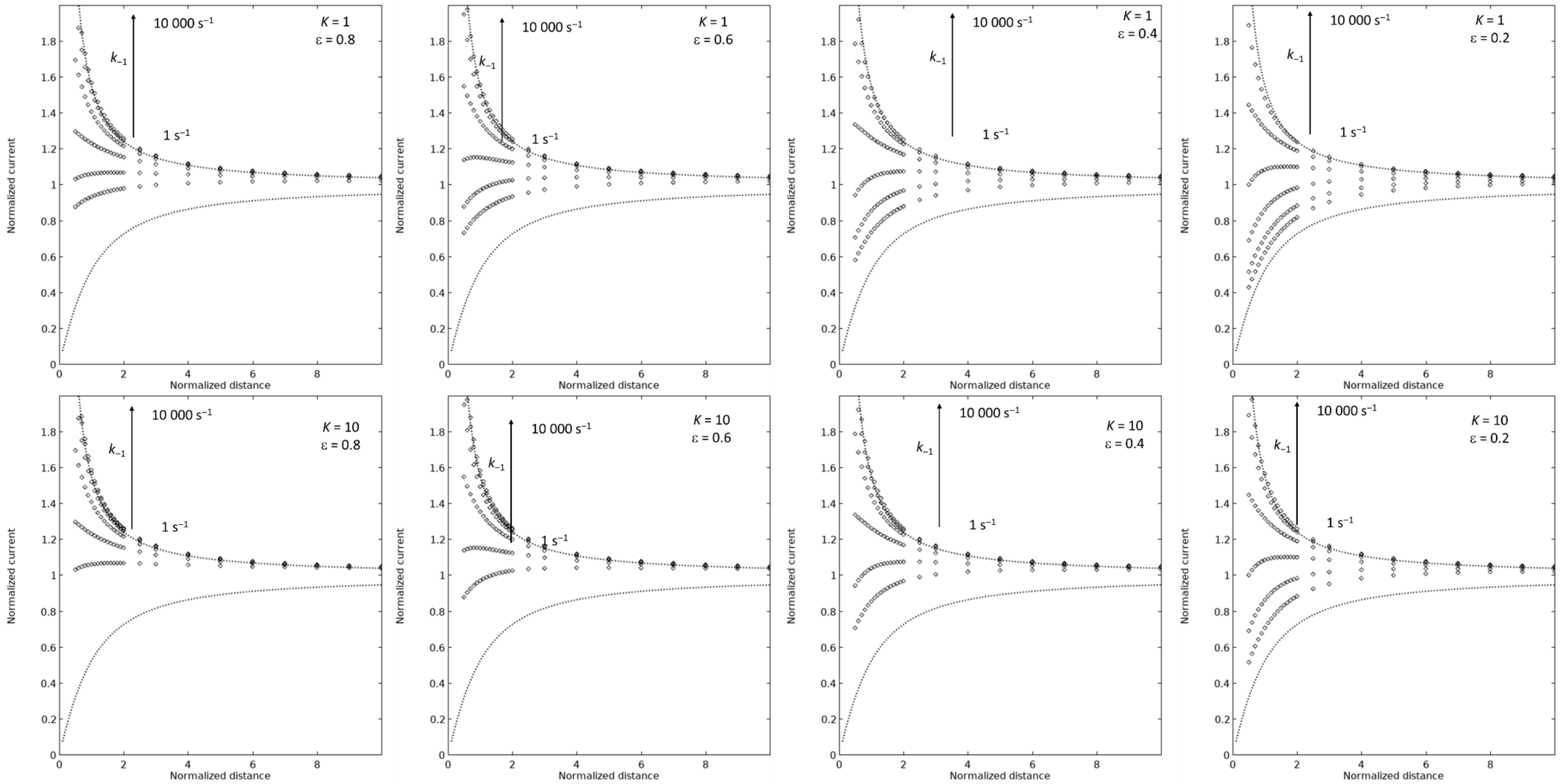
10 000 s⁻¹
k_{-1}
1 s⁻¹
K = 1
ε = 0.8
ε = 0.6
ε = 0.4
ε = 0.2
K = 10
Normalized current
Normalized distance

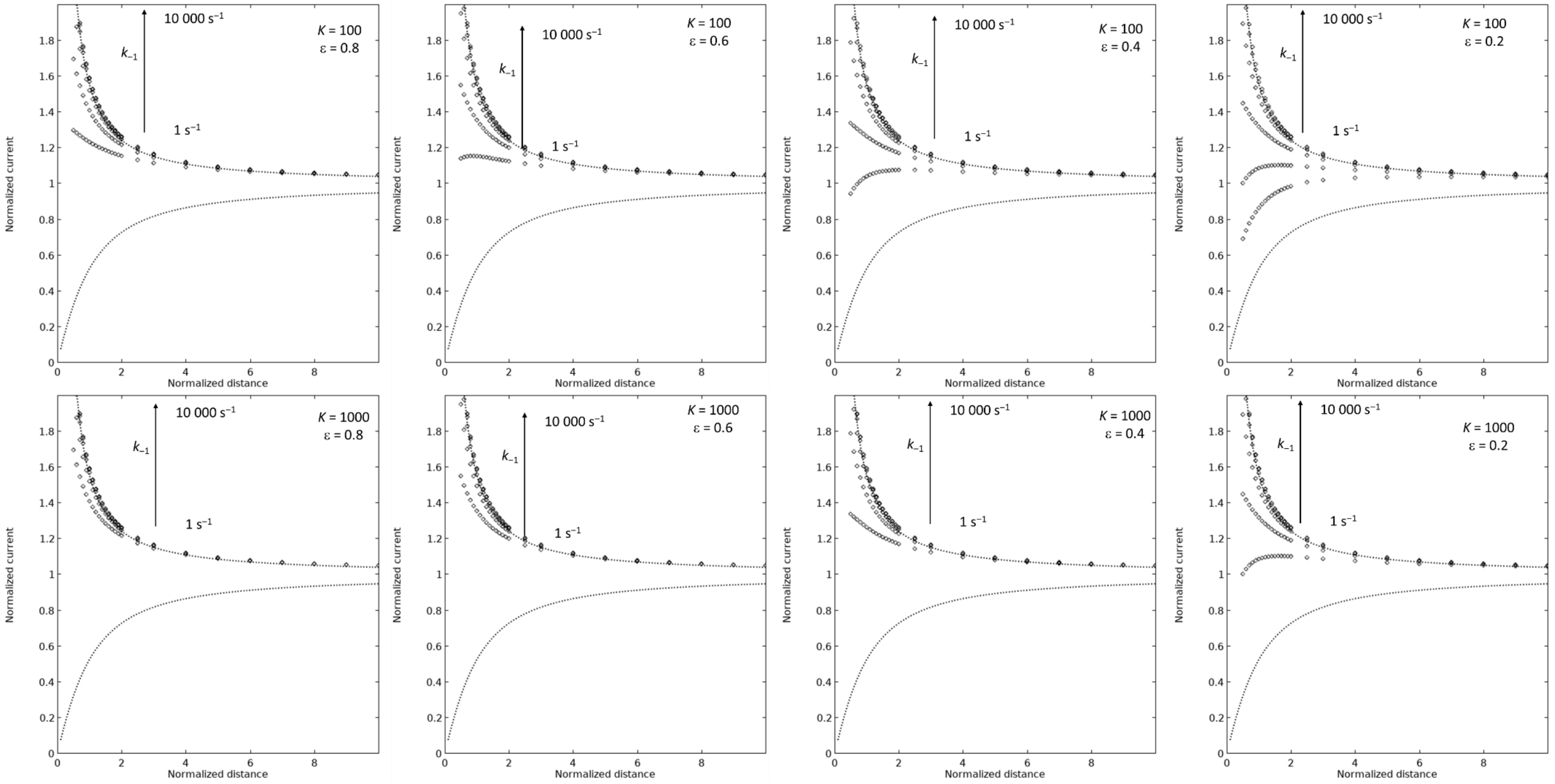

Figure S4. Simulated approach curves for electrode porosity *ε* from 0.2 to 0.8, partition coefficient *K* from 1 to 1000 and apparent transfer rate from gas phase to aqueous phase from 1 to 10 000 s^{-1}. Diffusion coefficient of oxygen in the gas phase is utilized for excess oxygen absorbed in the catalyst layer. Absorbed oxygen and electrolyte share the same space.

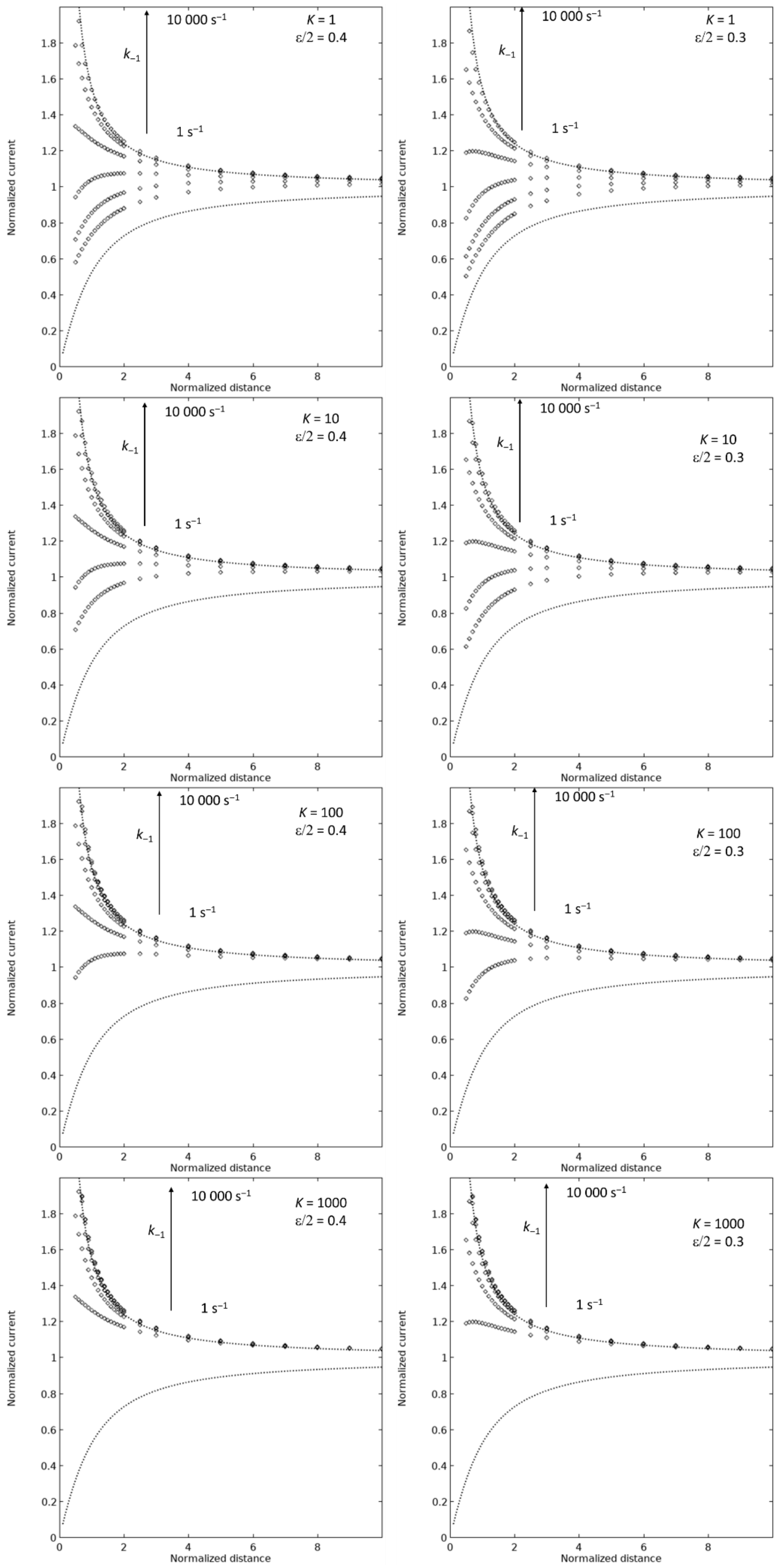

Figure S5. Simulated approach curves for electrode porosity ε from 0.2 to 0.8, partition coefficient K from 1 to 1000 and apparent transfer rate from gas phase to aqueous phase from 1 to 10 000 s^{-1}. Diffusion coefficient of oxygen in the aqueous gas is utilized for excess oxygen absorbed in the catalyst layer. The volume fraction of the electrolyte phase is half the porosity of the electrode, and volume fraction of the gas phase is equal to the volume fraction of the electrolyte.

Empirical equations for the theoretical positive and negative feedback response

Theoretical negative feedback (NI_T) is obtained with the empirical equation S7:

$$NI_T(L,\mathrm{R}_g) = \left[\frac{2.08}{\mathrm{R}_g{}^{0.358}}\left(L - \frac{0.145}{\mathrm{R}_g}\right) + 1.585\right] \times \left[\frac{2.08}{\mathrm{R}_g{}^{0.358}}\left(L + 0.0023\mathrm{R}_g\right) + 1.57 + \frac{\ln \mathrm{R}_g}{L} + \frac{2}{\pi \mathrm{R}_g}\ln\left(1 + \frac{\pi \mathrm{R}_g}{2L}\right)\right]^{-1} \qquad (S7)$$

where R_g (8 in this work) is the fraction of UME glass sheath radius over the electrode radius (*a*). Theoretical positive feedback (PI_T) is calculated with the empirical equation 2, containing an additional dimensionless kinetics parameter, κ:

$$PI_T(L,\mathrm{R}_g,\kappa) = GI_T\left(L + \frac{1}{\kappa},\mathrm{R}_g\right) + \frac{NI_T(L,\mathrm{R}_g) - 1}{(1 + 2.47\mathrm{R}_g{}^{0.31}L\kappa)(1 + L^{0.006\mathrm{R}_g+0.113}\kappa^{-0.0236\mathrm{R}_g+0.91})} \qquad (S8)$$

where $\kappa = ka/D$, *k* is the first order irreversible kinetics constant, *D* is the oxygen diffusion coefficient (1.9×10^{-5} cm^2/s), and $GI_T(L,\mathrm{R}_g)$ is defined as:

$$GI_T(L,\mathrm{R}_g) = \alpha(\mathrm{R}_g) + \frac{1}{\beta(\mathrm{R}_g)}\frac{\pi}{4ArcTanL} + \left(1 - \alpha(\mathrm{R}_g) - \frac{1}{2\beta(\mathrm{R}_g)}\right)\frac{2}{\pi}ArcTanL \qquad (S9)$$

where α and β are obtained as follows:

$$\alpha(\mathrm{R}_g) = ln2 + ln2\left(1 - \frac{2}{\pi}ArcCos\frac{1}{\mathrm{R}_g}\right) - ln2\left[1 - \left(\frac{2}{\pi}ArcCos\frac{1}{\mathrm{R}_g}\right)^2\right] \qquad (S10)$$

and

$$\beta(\mathrm{R}_g) = 1 + 0.639\left(1 - \frac{2}{\pi}ArcCos\frac{1}{\mathrm{R}_g}\right) - 0.186\left[1 - \left(\frac{2}{\pi}ArcCos\frac{1}{\mathrm{R}_g}\right)^2\right] \qquad (S11).$$

Cyclic voltammetry

Figure S6 a and b show the bulk solution cyclic voltammetry (CV) for basic (KOH 0.1M) and acidic (H_2SO_4 0.5M) media respectively. The potential range for CV was selected in order to observe the solvated oxygen reduction current . By sweeping the tip potential towards the negative values oxygen reduction current (absolute value) increased gradually and reached a maximum after which a plateau was observed until the commencement of hydrogen evolution reaction. The tip bias potential for approach curves and the substrate potential for oxygen depletion experiment in both media were selected from the potential window in which the plateau was observed (-0.6 V or -0.5 V was selected for basic and -0.1 V was selected for acidic media).

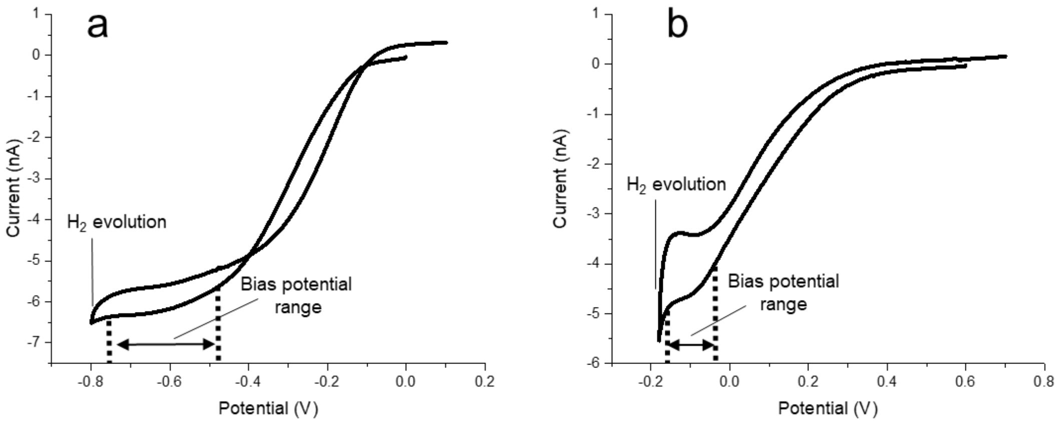

Figure S6. The bulk solution cyclic voltammetry a) for basic (KOH 0.1M) and b) for acidic (H_2SO_4 0.5M) media. The tip bias potential for approach curves and the substrate potential for oxygen depletion experiment in both media were selected from the potential window in which the plateau was observed.

Supplementary references

[1] M. Tavakkoli, E. Flahaut, P. Peljo, J. Sainio, F. Davodi, E. V Lobiak, K. Mustonen, E. I. Kauppinen, *ACS Catal.* **2020**, *10*, 4647–4658.

[2] P. Kanninen, B. Eriksson, F. Davodi, M. E. M. Buan, O. Sorsa, T. Kallio, R. W. Lindström, *Electrochim. Acta* **2020**, *332*, 1–11.

[3] C. Lefrou, R. Cornut, *ChemPhysChem* **2010**, *11*, 547–556.
